# Advancements in understanding the relationship between *Akkermansia muciniphila* and the development of ulcerative colitis

**DOI:** 10.3389/fped.2025.1673156

**Published:** 2025-09-26

**Authors:** Nie Xu, Xiaoping Xiao, Ying Ma, Jing Jing, ShiCheng Jiang, KeYong Luo, Ying Li, Miao Duan

**Affiliations:** ^1^ Department of Neonate, The Third Affiliated Hospital of Zunyi Medical University (The First People's Hospital of Zunyi), Zunyi, Guizhou, China; ^2^ Department of Pediatric, The Third Affiliated Hospital Zunyi Medical University (The First People's Hospital of Zunyi), Zunyi, Guizhou, China

**Keywords:** *Akkermansia muciniphila*, ulcerative colitis, intestinal barrie, immune regulation, strain specificity, microecological therapy

## Abstract

Ulcerative colitis (UC) is a chronic inflammatory bowel disease characterized by severe intestinal barrier dysfunction and immune dysregulation in patients, with limited clinical treatment options. Recent research highlights the important role of the gut bacterium *Akkermansia muciniphila (AKK)* in both the development and management of UC. *AKK* supports the integrity of the intestinal barrier by metabolizing mucins, enhances the production of tight junction proteins (Occludin-1/ZO-1), and influences immune responses by shifting macrophage polarization from M1 to M2, suppressing pro-inflammatory cytokines (IL-1β、IL-6、MCP-1), and activating anti-inflammatory pathways (SCFAs-SLC52A2/FFAR2、AhR). Clinical data indicate that the abundance of *AKK* in the intestines of patients with UC is significantly reduced, and this decrease is positively correlated with disease activity and relapse rates. Animal studies have demonstrated that adding *AKK* can restore the thickness of the mucus layer, lower inflammation scores, and improve the composition of gut microbiota. Importantly, the probiotic effects of *AKK* vary by strain; for instance, strain FSDLZ36M5 notably reduces colitis symptoms, while FSDLZ20M4 may worsen inflammation. These findings suggest that *AKK* or its metabolites, such as short-chain fatty acid(SCFAs), hold promise as therapeutic targets for the microbiota in patients with UC.Nonetheless, additional research on strain selection and clinical application is essential to refine treatment strategies. This article will review the correlation between the pathogenesis of *AKK* and UC, and explore the potential application value of *AKK* as a probiotic in children with UC, providing new insights for the prevention and treatment in patients with UC.

## Introduction

Ulcerative colitis (UC) is a gastrointestinal disease characterized by non-specific chronic inflammation of the colonic mucosa and submucosa, with an unclear etiology, and is classified as a type of inflammatory bowel disease (IBD). The clinical symptoms of UC include persistent diarrhea, abdominal pain, and rectal bleeding, with chronic inflammation typically confined to the colonic mucosal layer (1). During acute exacerbations, systemic toxic symptoms may occur, leading to severe complications, high mortality rates, and poor prognosis. Due to the long course of the disease, frequent relapses, and the lack of effective treatment methods, complete recovery is difficult. In children, the disease can not only cause abdominal pain, diarrhea, hematochezia, and extraintestinal symptoms, but may also lead to growth and developmental disorders as well as changes in mental status, with its incidence rising globally (2, 3). However, to date, there are no definitive effective preventive or therapeutic measures for this disease in either adults or children.

The exact mechanisms behind UC are not fully understood, but growing evidence suggests that gut microbiota and their byproducts significantly contribute to the disease's development (4). Additionally, both the initial appearance and subsequent flare-ups of UC are closely linked to the gut microbiome (5). Healthy gut bacteria support normal physiological processes in children by regulating immune responses, aiding metabolism, enhancing digestion and nutrient absorption, and counteracting harmful pathogens (5). Consequently, research into native human gut probiotics has opened new avenues for disease treatment. Notably, Bifidobacterium and Lactobacillus have emerged as well-established therapeutic probiotics available today (6). Research has also highlighted *Akkermansia muciniphila (AKK)*, a Gram-negative, oval bacterium isolated from human feces, which uses mucin for its carbon, nitrogen, and energy needs (7). Since the discovery of this unique bacterium in 2004, *AKK* has been extensively studied. It is found in breast milk and various parts of the digestive system, with the highest abundance in the colonic mucosal layer (8). It is considered a promising “next-generation beneficial microbe” (9).

### The biological characteristics of *AKK*

*AKK* is an anaerobic bacterium that thrives on mucus and is integral to the gastrointestinal microbiome, especially in mammalian intestines, being the sole representative of the Verrucomicrobia phylum. It relies exclusively on mucin for its carbon and nitrogen needs, allowing it to flourish within the intestinal mucosal layer. This distinctive metabolic trait positions *AKK* as vital for supporting intestinal health and balance. It is essential for preserving the function of the intestinal barrier, modulating immune responses, and ensuring metabolic stability (10). A key feature of *AKK* is its reliance on mucin for growth, which aids in the regeneration of the intestinal mucus layer. In healthy adults, it comprises about 1% to 4% of the total bacterial community in fecal samples, and its levels are inversely related to various inflammatory conditions, indicating a potential protective effect on gut health and overall physiological function by reinforcing the intestinal barrier (11, 12). Furthermore, *AKK* is known to generate short-chain fatty acids (SCFAs), which are vital for host metabolism and inflammatory processes (13). Research has also demonstrated that this bacterium bolsters the integrity of the intestinal epithelial barrier, solidifying its role as a beneficial component of the gut microbiota (10). It is important to note that the specific characteristics of different *AKK* strains may lead to varying health outcomes, suggesting that not all strains will provide the same probiotic benefits. This highlights the importance of careful strain selection and assessment for therapeutic uses, indicating that the probiotic effects of *AKK* are likely dependent on the specific strain (14). As investigations into *AKK* progress, it remains a significant contributor to gut microbiota composition and a promising candidate for future probiotic treatments aimed at enhancing metabolic health and maintaining gut ecosystem balance (15).

### Changes of *AKK* in UC

**Figure 1:**
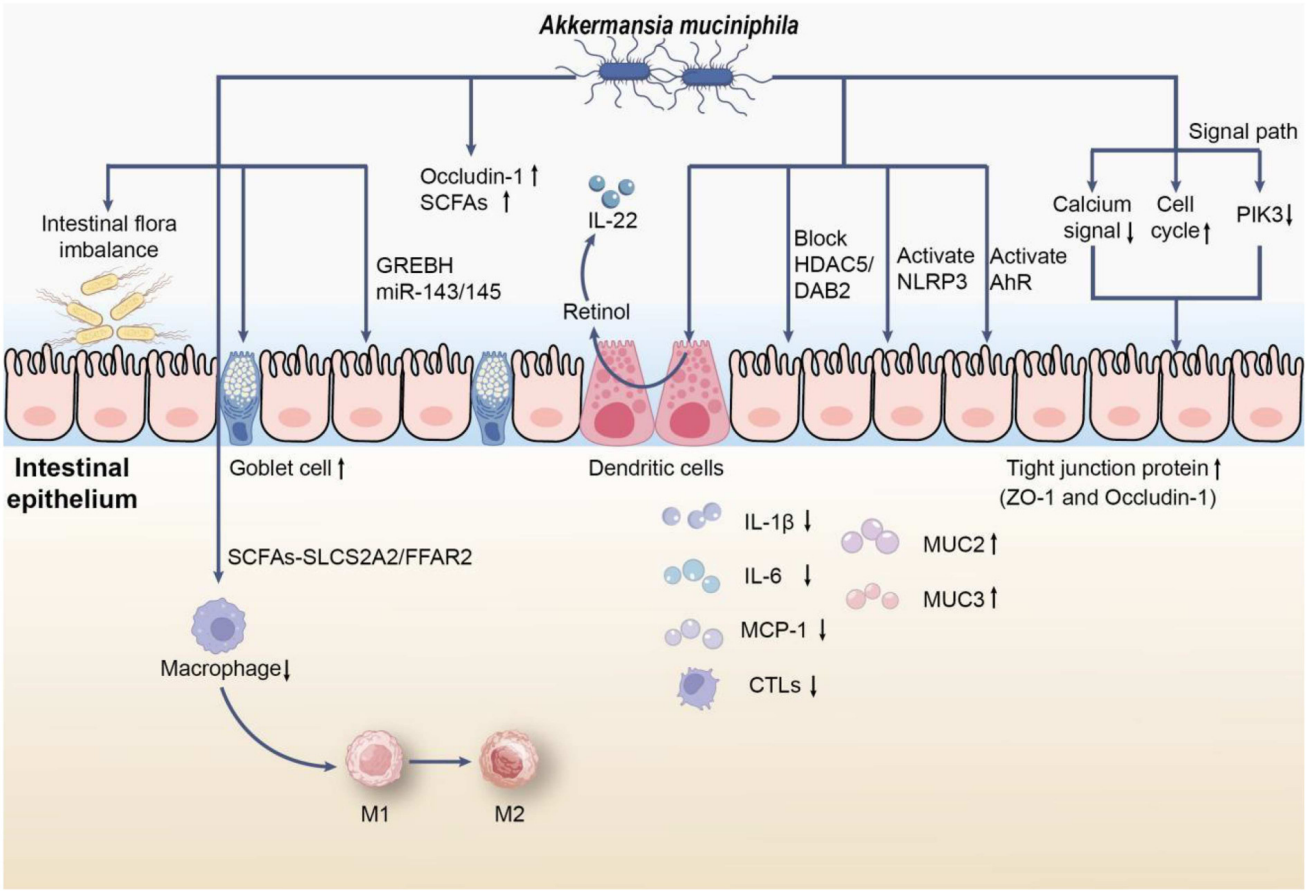
The role of *AKK* in UC. First, *AKK* may reduce colonic infiltrating macrophages through the SCFAs-SLC52A2/FFAR2 pathway and stimulate macrophage polarization from M1 to M2 *in vivo*, thereby alleviating the inflammatory response in mice with UC. In addition, *AKK* promotes mucin production by inducing an increase in the number and density of goblet cells, restoring the thickness of the mucus layer; simultaneously, its membrane proteins alleviate intestinal inflammatory stress via CREBH and miR-143/145. Moreover, *AKK* upregulates the expression of Occludin-1 and short-chain fatty acids(SCFAs) receptors, which can also promote the synthesis of retinoic acid in dendritic cells to regulate IL-22 activity and improve colonic damage in mice with UC by blocking the macrophage pro-inflammatory phenotype transition mediated by the HDAC5/DAB2 axis. On the other hand, *AKK* may reduce apoptosis by activating NLRP3 or AhR signaling pathways, or by downregulating the expression of calcium signaling pathway genes or upregulating cell cycle signaling pathway genes, or by alleviating intestinal epithelial cell inflammation through inhibiting the expression of upstream receptor genes of PI3K. Notably, the supplementation of *AKK* promotes the expression of tight junction proteins (such as ZO-1 and Occludin-1) and reduces colonic infiltrating cytotoxic T lymphocytes (CTLs), lowering the levels of pro-inflammatory cytokines (such as IL-1β, IL-6, and MCP-1), while promoting the expression of mucins (such as MUC2 and MUC3), further alleviating the inflammatory response.

*AKK* is crucial to the gut microbiome and has attracted considerable interest in the study of UC. Research conducted by Qian K. et al. revealed a notable decrease in *AKK* levels among individuals with UC (16). This decline is especially pronounced in those experiencing active UC, where *AKK* levels in the colon, cecum, transverse colon, left colon, and rectum are significantly lower (17). Such a reduction may contribute to the impairment of the intestinal mucosal barrier and the intensification of inflammatory reactions (18). Located within the intestinal mucus layer, *AKK* is vital for preserving gut homeostasis and structural integrity (Figure 1). Studies indicate that *AKK*, recognized as a mucin-degrading bacterium, may be instrumental in fostering the development of epithelial cells mediated by intestinal stem cells and in sustaining intestinal balance (19). Although *AKK* degrades mucin, animal experiments have shown that it can increase the production of mucin by enhancing the number and density of goblet cells in mice induced by a high-fat diet, thereby restoring the thickness of the mucus layer and strengthening the intestinal barrier (20). According to Wade H. et al., *AKK* and its membrane proteins mitigate intestinal inflammatory stress and enhance the healing of intestinal epithelial wounds via CREBH and miR-143/145 (21). In patients with UC, the decline in *AKK* correlates with intestinal barrier damage, evidenced by reduced mucin expression (notably MUC2), which is essential for the protective function of the epithelial barrier in the colon (22). This change in function may result in heightened intestinal permeability and inflammatory responses, characteristic of UC (16, 17).

Zhang L et al. demonstrated that *AKK* mitigated pathological damage in the colonic tissues of mice, reduced oxidative stress and inflammation, increased the levels of Occludin-1 and short-chain fatty acids(SCFAs) receptors, and promoted the transition of macrophages from the M1 to M2 phenotype *in vivo*. Following the silencing of FFAR2, the beneficial effects of *AKK* on cell viability and the M1 to M2 macrophage shift, along with its suppression of oxidative stress, inflammation, apoptosis, metabolic disorders, and necroptosis, were reinstated. This suggests that *AKK* may influence macrophage polarization via the SCFAs-SLC52A2/FFAR2 pathway, thus alleviating the inflammatory response in mice with dextran sulfate sodium (DSS)-induced UC (23). Additional research indicates that *AKK* improves colonic damage in DSS-induced UC by inhibiting the pro-inflammatory phenotypic shift in macrophages mediated by the HDAC5/DAB2 axis (24). Furthermore, *AKK* enhances retinoic acid production in dendritic cells, which helps regulate IL-22 activity and reduce colitis symptoms in mice (25). Moreover, Qu S et al. found that *AKK* can ease DSS-induced acute colitis through the activation of NLRP3 (26). Additionally, *AKK* has the ability to activate AhR signaling by influencing tryptophan metabolism, leading to a decrease in colonic inflammation (27).

Research indicates that a decrease in *AKK* correlates with elevated levels of pro-inflammatory cytokines, suggesting its significant role in regulating immune responses within the gut (13, 28). Dietary inclusion of *AKK* has been found to strengthen the intestinal barrier by enhancing the production of tight junction proteins like ZO-1 and Occludin-1, which are essential for preserving epithelial cell integrity (29). Luo Y and colleagues discovered that *AKK* might mitigate cell apoptosis by downregulating critical genes in the calcium signaling pathway or upregulating those in the cell cycle signaling pathway. Additionally, it may reduce inflammation in intestinal epithelial cells by inhibiting the expression of upstream receptor genes in the PI3K pathway (30). Moreover, *AKK* supplementation has been shown to lower inflammatory markers, offering protection against colitis induced by dextran sulfate sodium (DSS) (18). Importantly, the health advantages of *AKK* may also stem from its metabolites, particularly short-chain fatty acids (SCFAs), which could significantly influence the host's metabolic health and inflammatory conditions (31). *AKK* appears to be instrumental in modulating immune responses and enhancing gut health, thereby contributing to reduced inflammation in UC (32). Furthermore, evidence suggests that *AKK* can restore gut microbiota diversity, increasing the prevalence of beneficial bacteria like Bacteroides and Lactobacillus, which may further bolster its protective effects against colonic inflammation (33). Another significant discovery relates to the strain-specific effects of *AKK*, revealing that different strains can have distinct impacts on gut health. Liu Q and associates found that among three isolated human *AKK* strains (FSDLZ39M14, FSDLZ36M5, and FSDLZ20M4) and the *AKK* type strain ATCC BAA-835, only FSDLZ36M5 exhibited notable protective effects against UC by increasing colon length, decreasing intestinal permeability, and enhancing the expression of anti-inflammatory cytokines. The other strains (FSDLZ39M14, ATCC BAA-835, and FSDLZ20M4) did not demonstrate these benefits, with FSDLZ20M4 showing a tendency to worsen inflammation according to various indicators (34). This highlights the necessity of identifying specific strains with therapeutic potential for UC management and offers genetic targets for the efficient and rapid screening of *AKK* strains that may alleviate UC symptoms.

### The potential therapeutic role of *AKK* in UC

*AKK* has become a key candidate for treating UC due to its vital function in supporting gut health and regulating inflammation. Research indicates that adding *AKK* can lead to notable improvements in the Disease Activity Index (DAI), colon length, and organ index (35). Furthermore, *AKK* has been shown to decrease the presence of macrophages and cytotoxic T lymphocytes (CTLs) in the colon, which helps to mitigate inflammatory reactions (36). A study by Liu F et al. revealed that *AKK* supplementation resulted in lower levels of pro-inflammatory cytokines, including IL-1β, IL-6, and MCP-1. This effect may be linked to *AKK's* involvement in host immune signaling pathways, particularly through members of the nucleotide-binding oligomerization domain-like receptor family, such as NLRP3, which is known to play a role in inflammation regulation (32). In a model of UC induced by dextran sulfate sodium (DSS), *AKK* supplementation improved intestinal barrier function, decreased the infiltration of inflammatory cells, and increased the expression of mucins like MUC2 and MUC3, which are essential for maintaining intestinal integrity (37). This suggests that enhancing *AKK* levels through diet or probiotics could be a viable approach for UC treatment. Additionally, the damage to the mucus layer that *AKK* relies on is thought to significantly contribute to UC's development; thus, its involvement in mucin degradation and metabolic functions may be critical for restoring gut balance (38). While the therapeutic potential of *AKK* is encouraging, further clinical trials are needed to assess its effectiveness and safety in patients with UC. More in-depth studies on specific strains and their mechanisms are crucial for fully understanding how to incorporate *AKK* into treatment plans for UC and other gastrointestinal issues (39).

### The potential application value of *AKK* as a probiotic in children with UC

Current research on the role of *AKK* in IBD primarily focuses on adult and animal models. In pediatric populations, only two studies explicitly mention the association between this bacterium and IBD**.** Therefore, this section will explore the potential application value of *AKK* as a probiotic in children with UC.

In patients with UC, the reduction of *AKK* may be closely related to disease relapse and progression (40). This reduction could lead to a higher likelihood of disease recurrence, prompting studies to suggest that changes in the abundance of *AKK* may serve as a biomarker for predicting disease relapse (41). In pediatric populations, the gut microbiota is still developing, making it more susceptible to dysbiosis. The ability of *AKK* to modulate microbial composition and enhance mucosal immunity may be particularly beneficial, leading to the inference that this pathogenic mechanism is also applicable to pediatric patients. Furthermore, *AKK* has garnered attention for its potential protective role in gut health, especially among children with UC. It is believed that *AKK* can renew the mucus layer in the gut and enhance barrier integrity, which is often compromised in patients with UC (41). Therefore, enhancing mucosal barrier function is crucial, particularly for children, as further exacerbation of UC may have lasting impacts on their growth and development.Experimental studies have indicated that *AKK* supplementation can lead to positive outcomes, such as decreased inflammatory cell infiltration and enhanced mucin production (notably MUC2 and MUC3) in the intestinal lining (26). The administration of *AKK* seems to alleviate colitis symptoms by bolstering intestinal barrier function and mitigating inflammation (11), underscoring its potential as a treatment option for pediatric UC. Importantly, the characteristics of intestinal inflammation include immune cell infiltration and damage to goblet cells (41). The presence of sufficient *AKK* in the gut may counteract these issues, providing a supplementary strategy for managing pediatric UC. Therefore, *AKK* and other dietary or therapeutic interventions, such as prebiotics or fecal microbiota transplantation (FMT), are worth exploring to optimize treatment outcomes (42). Furthermore, understanding the phylogenetic variation and phenotypic diversity of *AKK* may offer further insights into personalized treatment approaches for pediatric UC. Different strains of *AKK* exhibit unique characteristics that may influence their effectiveness in modulating the gut environment and alleviating UC symptoms (43). Despite these promising findings, further research is needed to elucidate the protective role of *AKK* in pediatric UC and to establish standardized protocols for its clinical application. Large-scale, randomized controlled trials are essential to validate its efficacy, safety, and long-term benefits in pediatric populations.

In conclusion, *AKK* is a key element in understanding the microbiota's role in pediatric UC, with its capacity to influence gut health and immune responses presenting a promising path for therapeutic development. Ongoing studies are needed to clarify the specific mechanisms and best practices for employing this bacterium in treating pediatric UC, emphasizing the importance of further investigation into the therapeutic possibilities of probiotics like *AKK,* Especially in the group of children with UC.

## Conclusion

While current research has suggested that *AKK* may play a significant role in UC, the exact mechanisms involved remain to be explored. Future studies might concentrate on: 1. how *AKK* interacts with the intestinal immune system; 2. the particular impact of *AKK* in ulcerative colitis among children; 3. the creation of innovative therapeutic strategies based on *AKK*, including probiotic products or their metabolites (44, 45).

**Figure 2:**
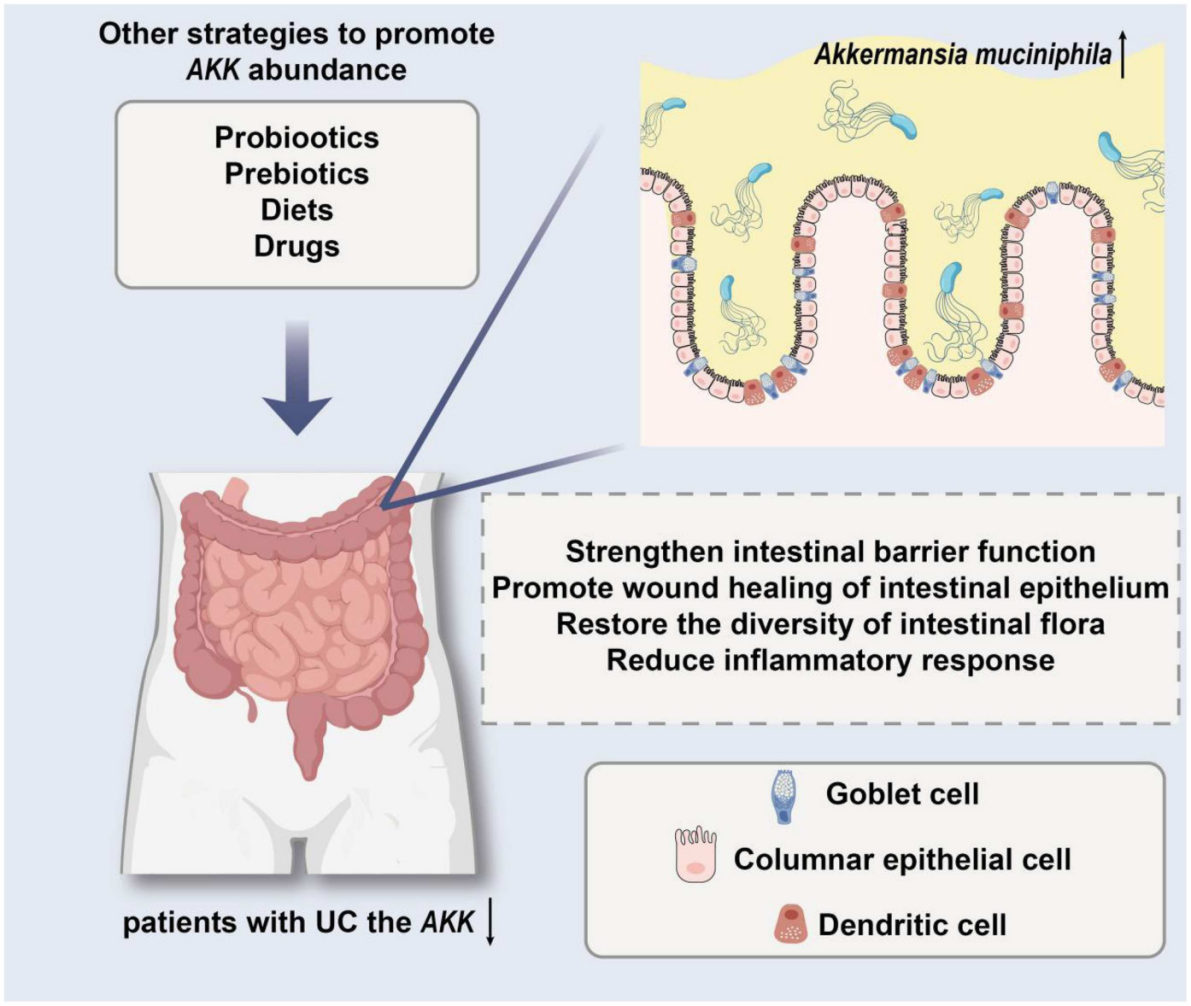
The model demonstrates the protective effect of targeting *AKK* in the treatment of ulcerative colitis (UC).

To conclude, *AKK* is a crucial component of gut microbiota that significantly influences the development of UC. A decrease in its levels correlates strongly with the severity and relapse of the condition, suggesting that introducing *AKK* could be a promising treatment approach. Ongoing studies are expected to clarify how it functions, offering fresh perspectives for preventing and managing UC (Figure 2).
